# Short‐time effect of intravitreal injections on retinal vascular oxygenation and vessel diameter in patients with diabetic macular oedema or neovascular age‐related macular degeneration

**DOI:** 10.1111/aos.14276

**Published:** 2019-10-25

**Authors:** Christoph Mitsch, Berthold Pemp, Andreas Pollreisz, Andreas Gleiss, Sonja Karst, Christoph Scholda, Stefan Sacu, Ursula Schmidt‐Erfurth

**Affiliations:** ^1^ Department of Ophthalmology and Optometry Medical University of Vienna Vienna Austria; ^2^ Center for Medical Statistics Informatics and Intelligent Systems Medical University of Vienna Vienna Austria

**Keywords:** anti‐VEGF, choroidal neovascularization, diabetic macular oedema, oximetry, retina

## Abstract

**Purpose:**

To investigate the short‐time effect of intravitreal injections (IVI) of the vascular endothelial growth factor inhibitors ranibizumab and aflibercept on retinal arterial and venous oxygen saturation (SO2a and SO2v), arteriovenous oxygen saturation difference (AVD) and vessel diameter (VDa and VDv) in patients with diabetic macular oedema (DME) and patients with choroidal neovascularization (CNV) due to age‐related macular degeneration.

**Methods:**

Uncontrolled prospective observational study in 100 eyes. Retinal vessel oxygen saturation and diameters were assessed using a retinal oximeter before and minutes after IVI of ranibizumab or aflibercept.

**Results:**

40 eyes with CNV and 34 eyes with DME were included in the analysis. At baseline, SO2a and SO2v were significantly higher in DME (p = 0.043 and p = 0.009, respectively). After IVI, SO2a significantly decreased in CNV and DME eyes by 2.6% (p = 0.016) and 4.6% (p = 0.002) and SO2v decreased by 14.0% (p = 0.004) and 12.4% (p = 0.017), respectively. However, a significant increase in AVD was only found in CNV (15.7%, p = 0.001). VDa decreased significantly only in DME by 5.7% (p = 0.010). No medication‐specific disease effect was found and vice versa.

**Conclusions:**

The observed changes can be interpreted as signs of increased metabolic demand during the physiological stress after an IVI. The abnormal arterial constriction and the abolished increase in AVD seen only in eyes with DME indicate an impairment of vascular autoregulation and oxygen distribution and a reduced neuroretinal metabolism in the diabetic retina with a significant impact on inner retinal oxygen consumption shortly after IVI.

## Introduction

Neovascular age‐related macular degeneration (AMD) and diabetic retinopathy (DR) are the most common causes of severe vision loss in the western world (Ambati et al. 2003; Yau et al. 2012). Diabetic macular oedema (DME) is primarily responsible for vision impairment in diabetic patients (Klein 2007; Nicholson & Schachat 2010; Soheilian et al. 2012). The inhibition of vascular endothelial growth factor (VEGF), an angiogenesis and vasopermeability factor prominently involved in the pathogenesis of DME and CNV, has proven to be an effective therapeutic approach and is widely used for treating both neovascular AMD and DME (Ambati et al. 2003; Nicholson & Schachat 2010; Schmidt‐Erfurth et al. 2014; Wells et al. 2016). Two anti‐VEGF agents for intravitreal injection (IVI) in pathologic eyes are currently approved for the treatment of CNV and DME: ranibizumab (Lucentis, Genentech, San Francisco, USA) and aflibercept (Eylea, Regeneron Pharmaceuticals, New York, USA).

Retinal oximetry has been developed to enable *in vivo*, non‐invasive oxygen saturation measurement in human retinal vessels and has provided new insights into disease mechanisms of different common eye diseases associated with vascular alterations. It was shown that oxygen extraction defined as arteriovenous difference in oxygen saturation (AV difference) is lowered in patients with diabetic retinopathy and further reduces with progression of the disease (Jørgensen et al. 2014). A reduced AV difference in retinal vessels was also described in eyes with CNV (Geirsdottir et al. 2014). Recently, a reduction in retinal venous oxygen saturation was shown in patients with central retinal vein occlusion (CRVO) which stayed lower during monthly anti‐VEGF treatment (Traustason et al. 2014).

Until now, no data about the instant effects of anti‐VEGF IVIs on retinal vessel oxygenation and AV difference in eyes with CNV and DME are available. Hence, the aim of this study was to assess the instant effects of IVIs on the oxygen saturation in retinal vessels of patients with CNV and DME.

## Materials and Methods

### Study procedures

This uncontrolled, prospective observational study was performed at the Department of Ophthalmology of the Medical University of Vienna. All the research and measurements adhered to the tenets of the Declaration of Helsinki. The study was approved by the local ethics committee and informed consent was obtained from all individuals after a detailed discussion of the nature and possible consequences of the study procedures.

We measured 100 consecutive eyes planned for an IVI of either ranibizumab (25 treated for DME and 25 for CNV) or aflibercept (25 treated for DME and 25 for CNV).

The inclusion criteria comprised the will to participate in this study documented by written and signed informed consent, the ability to fixate the external fixation light of the Oxymap fundus camera and the presence of either DME secondary to diabetes mellitus or active CNV on one or both eyes. Exclusion criteria were media opacities including advanced cataract or vitreous preretinal haemorrhage, the co‐presence of other than disease‐specific retinal pathologies, relevant fixation difficulties or any intravitreal treatment received <4 weeks earlier.

### Intravitreal medication

Intravitreal injections were administered under sterile conditions in an operating room. The procedure was performed in a standardized manner after topical anaesthesia with 0.5% oxybuprocaine and 1% lidocaine eyedrops, application of povidone–iodine to the bulbar conjunctiva, the fornices, eyelid margins and lashes, application of a sterile drape and insertion of a lid speculum. A volume of 0.05 ml containing 0.5 mg of ranibizumab or 2.0 mg of aflibercept was injected 3.5 to 4.0 mm posterior to the superotemporal limbus through the pars plana into the vitreous cavity using a 30‐gauge needle.

### Retinal oximetry

Automated retinal oximetry was performed using the Oxymap T1 (Oxymap Inc., Reykjavik, Iceland), a commercially available retinal oximetry device, as previously described (Geirsdottir et al. 2012). In short, it estimates retinal vessel oxygen saturation from fundus photographs taken simultaneously at different wavelengths by comparing the light absorbance of retinal vessels at a wavelength sensitive to oxygen saturation (600 nm) to a reference wavelength (570 nm). The proprietary software automatically selects retinal vessels on the images and calculates relative oxygen saturation values automatically adjusted for vessel diameters. Measurements using retinal oximetry have shown good repeatability for oxygen saturation and vessel calibres and good sensitivity to changes in oxygen saturation (Hardarson et al. 2006; Palsson et al. 2012)

Measurements were performed after pupil dilation immediately before and a short time after the IVI procedure. Mydriasis was achieved by instilling phenylephrine and tropicamide eyedrops 10 min before the pre‐IVI photography. The photographs were taken at a dim light setting at the same ward where the injection took place just two rooms away from the injection room. Before analysis, image quality was graded manually (1: excellent, 2: acceptable, 3: marginal, 4: insufficient quality) in term of focus, contrast and overall quality considering shadows, glare or other abnormalities, as recommended in the manufacturer protocol (Protocol for acquisition and analysis of Oxymap T1 oximetry images 2013). Eyes with image quality grading worse than 2/acceptable were excluded from the study. Analysis of the fundus photographs was performed using the supplied Oxymap analysis software V2.4.0, Rev. 6813 also following the standard manufacturer protocol. All vessel segments between two circles of 1.5 and 3 times the optic disc diameter centred at the optic disc were chosen for the automated analysis of the mean arterial and venous oxygen saturation and diameter.

### Statistical analysis

Continuous variables were described by medians and quartiles (interquartile range, IQR) or medians and ranges due to non‐symmetric distributions. Baseline values were compared using Wilcoxon's rank‐sum test. Negative saturation values occurred in 23 out of 2163 measurements and were set to zero. For each of the five outcomes, all measurements within an eye were averaged. Each outcome was log‐transformed to achieve approximately normally distributed conditional residuals. Effect estimates were back‐transformed to change ratios on the original scale. Mixed models were used to investigate the dependence of each averaged outcome on disease group and medication. The interaction of disease and medication was not included due to statistical insignificance for all outcomes. Each model included the respective log‐transformed baseline measurement, an indicator for left/right eye and the log‐transformed interval between measurements as fixed effects as well as a random patient effect to account for the ten patients who contributed both eyes.

Least‐squares means with 95% confidence intervals as well as contrasts and p‐values were reported based on these models. The p‐values were not corrected for testing multiple outcomes due to the exploratory character of the study.

## Results

### Patient characteristics

74 eyes of 64 patients were included in the analysis (CNV: 40 eyes, DME: 34 eyes). In 7 eyes, imaging after IVI was not possible and 19 eyes (5 CNV and 14 DME eyes) were excluded because of marginal or insufficient image quality of either the pre‐ or the postintervention image or both. Table 1 shows the baseline characteristics of the patient groups. All DME patients had clinically significant macular oedema.

**Table 1:** Patient demographics.

Pathology	DME		CNV		All
Treatment	Ranibizumab	Aflibercept	Ranibizumab	Aflibercept	
N (patients/eyes)	15/18	12/16	16/18	21/22	64/74
male/female	14/13		17/20		31/33
Median age (yrs) and range	66.5 (51.7–76.1), IQR [59.9, 72.4]		79.9 (55.2–92.8), IQR [71.0, 86.3]		72.7 (51.7–92.8), IQR [66.0, 80.9]
Proportion of eyes with image quality grade 2 (acceptable) before/after IVI	59%/79%		49% / 62%		54% / 70%

CNV = choroidal neovascularization, DME = diabetic macular oedema, IVI = intravitreal injection, IQR = interquartile range.

Image quality grades: 1: excellent, 2: acceptable, 3: marginal (excluded), 4: insufficient (excluded).

All DME patients also had signs of diabetic retinopathy. See Table 2 for the diabetes‐specific baseline characteristics of the DME subcohort including international clinical diabetic retinopathy severity grading (Wilkinson et al. 2003).

**Table 2:** Diabetes‐specific baseline characteristics of the diabetic macular oedema (DME) subcohort.

Treatment	Ranibizumab	Aflibercept	All DME
Diabetes type	100% Type 2	100% Type 2	100% Type 2
Mean glycated haemoglobin (%)	7.19 ± 0.64	8.25 ± 2.52	7.69 ± 1.73
Mean diabetes duration (years)	22.9 ± 12.4	15.7 ± 9.9	19.44 ± 11.83
Insulin‐dependent	8 (53%)	7 (58%)	15 (55%)
Duration of insulin treatment (years), if applicable	13.7 ± 12.1	13.9 ± 5.5	13.80 ± 8.55
Diabetic retinopathy staging			
Proliferative	4 (22%)	5 (31%)	9 (26%)
Severe	4 (22%)	3 (19%)	7 (21%)
Moderate	3 (17%)	2 (13%)	5 (15%)
Mild	5 (28%)	3 (19%)	8 (24%)
Unknown (not graded)	2 (11%)	3 (19%)	5 (15%)

### Measurement characteristics

Preinjection measurements were performed immediately before treatment. Postinjection measurements were performed shortly after the IVI procedure. Median time between injection and postinjection examination was 9 min (range 2–63, IQR 5, 11) for all eyes, 7 min (range 2–57, IQR 4.5, 9) for CNV eyes and 10 min (range 3–63, IQR 8, 13) for DME eyes.

The number of measured vessels in each patient varied between 2 and 12 arteries and 3 and 10 veins. The analysed vessel segments had a minimum diameter of 93 *μ*m and a minimum length of 465 *μ*m. For each eye, the same segments of the same retinal arteries and veins were analysed before and after IVI. Image quality after IVI only changed in a minority of patients (see Table 1).

### Baseline measurements

Table 3 shows arterial and venous oxygen saturation, AV difference and vessel diameters for CNV and DME eyes before IVI. Arterial and venous oxygen saturation was significantly higher in DME eyes than in CNV eyes (p = 0.043 and p = 0.009, respectively). Venous vessel diameter was similar between the two patient groups, whereas arterial diameter and AV difference were lower in diabetes patients but not significantly different from patients with AMD.

**Table 3:** Baseline measurements of retinal oximetry.

	DME	CNV	p‐Value*
Median arteriolar O2 saturation	97.5% IQR [96.1, 101.5]	92.9% IQR [92.9, 98.3]	**0.043**
Median venular O2 saturation	67.3% IQR [60.7, 71.4]	62.0% IQR [53.9,67.2]	**0.009**
Median AV difference	30.1% IQR [19.9, 65.9]	32.8% IQR [27.9, 40.7]	0.129
Median arteriolar diameter (*μ*m)	104.2 IQR [101.7, 116.2]	110.8 IQR [102.1, 122.6]	0.143
Median venular diameter (*μ*m)	141.6 IQR [130.0, 155.5]	140.9 IQR [132.9, 155.3]	0.909

AV difference = Arteriovenous difference in oxygen saturation, CNV = choroidal neovascularization, DME = diabetic macular oedema, IQR = interquartile range.

* p‐values not corrected for multiple testing, bold numbers indicate significant p‐values.

Table 4 shows the change ratios of the measured parameters in the two patient groups after IVI compared to baseline.

**Table 4:** Mean change ratio (post‐IVI divided by baseline) of all assessed outcome parameters in the different disease groups. AV difference: arteriovenous difference in oxygen saturation. For arterial diameter, the change ratio was significantly different between disease groups (p = 0.021).

Measurement	Disease	Change ratio	Confidence interval	p‐Value^†^
Arterial O2 saturation	CNV	0.97	(0.95, 0.99)	**0.0157**
	DME	0.95	(0.93, 0.98)	**0.0017**
Venous O2 saturation	CNV	0.86	(0.79, 0.94)	**0.0037**
	DME	0.88	(0.79, 0.97)	**0.0167**
AV difference	CNV	1.16	(1.08, 1.24)	**0.0009**
	DME	1.05	(0.97, 1.13)	0.1676
Arterial diameter	CNV	1.01	(0.98, 1.05)	0.4972
	DME	0.94	(0.91, 0.98)	**0.0101**
Venous diameter	CNV	1.02	(0.98, 1.06)	0.2208
	DME	1.00	(0.95, 1.04)	0.8984

^†^ p‐values not corrected for multiple testing, bold numbers indicate significant p‐values.

### Change in arterial oxygen saturation

**Figure 1:**
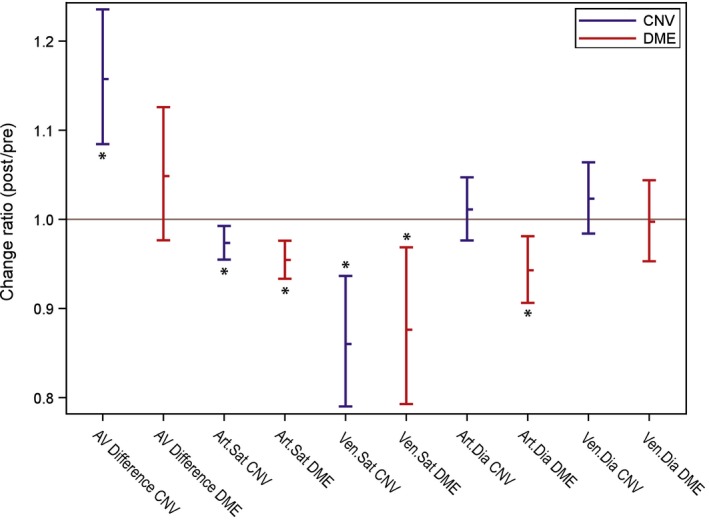
Change ratios of arteriovenous difference in oxygen saturation (AV Difference), arterial and venous saturation (Art.Sat. and Ven.Sat.) and diameter (Art.Dia. and Ven.Dia.) for CNV (choroidal neovascularization, blue) and DME (diabetic macular oedema, red) eyes presented as least‐squares means and 95% confidence intervals. The asterisk signs indicate statistically significant change ratios (p < 0.05).

After IVI the arterial oxygen saturation significantly decreased by 2.6% in CNV eyes (p = 0.0157) and by 4.6% in DME eyes (p = 0.0017) (Table 4). Figure 1 shows the proportional differences in CNV and DME eyes. The mean relative change in arterial oxygenation was nearly two times higher in diabetes patients but showed no statistically significant difference to patients with CNV (p = 0.193). There was also no significant difference between eyes treated with ranibizumab compared to eyes treated with aflibercept (p = 0.269). The statistical interaction between disease (CNV, DME) and medication (ranibizumab, aflibercept) was not statistically significant (p = 0.591), thus no medication‐specific disease effect was found and vice versa.

### Change in venous oxygen saturation

Venous oxygen saturation significantly decreased on average by 14.0% in CNV eyes (p = 0.0037) and by 12.4% in DME eyes (p = 0.0167) (Table 4, Fig. 1). The mean saturation change was somewhat larger in CNV than in DME, but the difference was not statistically significant (p = 0.765). The small difference between the treatment groups was also not statistically significant (p = 0.097). The statistical interaction between disease and medication was not statistically significant (p = 0.748); thus, no medication‐specific disease effect was found and vice versa.

### Change in AV difference

Different changes in AV difference after IVI were observed in CNV and DME eyes. On average, AV difference increased significantly by 15.7% in CNV eyes (p = 0.0009) whereas the small change of 4.9% in DME eyes was statistically not significant (p = 0.168) (Table 4, Fig. 1). The mean change in AV difference was three times higher in CNV eyes than in DME eyes but the comparison between patient groups showed no statistical significance (p = 0.055). The change in AV difference was not different between treatment groups (p = 0.375). The statistical interaction between pathology and medication was not statistically significant (p = 0.478); thus, no medication‐specific disease effect was found and vice versa.

### Change in arterial diameter

After injection, the mean arterial diameter in CNV eyes increased only by 1.1%, but this change was not statistically significant (p = 0.497). In DME eyes, however, mean arterial diameter decreased significantly by 5.7% (p = 0.010) (Table 4, Fig. 1). This change in arterial diameters of DME patients was also statistically significant compared to CNV patients (p = 0.021). Changes in eyes treated with ranibizumab were not different from eyes treated with aflibercept (p = 0.940). The statistical interaction between pathology and medication was not statistically significant (p = 0.608); thus, no medication‐specific disease effect was found and vice versa.

### Change in venous diameter

After IVI venous diameters in eyes with CNV tended to increase on average by 2.3%, but this change was not statistically significant (p = 0.221) (Table 4, Fig. 1). Venous diameters in eyes with DME were nearly unchanged (−0.3%; p = 0.898). The mean change in vein diameter was not different between patient (p = 0.377) or treatment groups (p = 0.655). The statistical interaction between pathology and medication was not statistically significant (p = 0.778); thus, no medication‐specific disease effect was found and vice versa.

## Discussion

This is the first study to evaluate oxygen saturation in retinal vessels, AV difference and vessel diameters on the same day shortly before and after IVI of anti‐VEGF agents in eyes with neovascular AMD or DME.

Previous long‐term examinations comparing dual‐wavelength oximetry before and months after IVI found no effect of anti‐VEGF therapy on retinal vessel diameters and oxygenation in patients with DME (Bek & Jørgensen 2016) and an increase in oxygen saturation of retinal arteries only in a subgroup of CNV patients not responding to treatment (Jakobsen et al. 2019). The baseline oxygenation values we measured in retinal arteries and veins of CNV eyes were comparable to values in a previous study using the same device, in which they were similar to those of healthy eyes (Geirsdottir et al. 2014). In eyes with DME, we found significantly higher oxygenation levels in retinal arteries and veins, which is also consistent with previously published data (Hammer et al. 2009; Hardarson & Stefánsson 2012; Khoobehi et al. 2013; Jørgensen et al. 2014; Bek & Jørgensen 2016). The increased blood oxygenation levels in retinal veins have been attributed to several disease‐specific changes in diabetic retinopathy leading to a reduced oxygen consumption in retinal tissue: Predominantly, diabetes‐induced microvascular changes such as basement membrane thickening, reduced capillary density and shunt vessel formation may lead to reduced tissue perfusion and hence limit oxygen uptake and distribution in the retina (Pemp & Schmetterer 2008; Czakó et al. 2018). In addition, neuroretinal tissue loss mainly of the inner retinal layers, which increases with diabetes duration (Pemp et al. 2018), could also reduce retinal oxygen consumption. Increased total retinal blood flow in diabetic retinopathy and the higher affinity of oxygen to glycosylated haemoglobin, which is elevated in diabetes patients, may as well account for an oxygenation increase in retinal veins and also in retinal arteries (Bek 2013; Klefter et al. 2015). Besides, a counter‐current mechanism of diffusional oxygen exchange from arteries to adjacent veins has been proposed, which could also gradually increase arterial oxygenation with increased venous oxygenation, possibly already at the level of the central retinal artery (Hardarson & Stefánsson 2012).

AV difference at baseline was non‐significantly lower in our patients with DME. However, previous studies comparing patients to healthy controls have shown a reduced AV difference in eyes with diabetic retinopathy (Jørgensen et al. 2014; Fondi et al. 2017) as well as in elderly patients with exudative AMD (Geirsdottir et al. 2014) indicating reduced basal retinal oxygen consumption in both pathologies.

Oximetry showed a significant reduction in oxygen saturation of retinal arteries and even more pronounced in retinal veins shortly after IVI in both groups. A reduction in venous oxygen saturation alongside an increase in AVD would indicate an increase in oxygen consumption after an intervention. This can be driven either by an increased metabolism or by reduced blood flow. The reduction in arterial oxygen saturation is an unexpected finding but could be due to the aforementioned diffusional oxygen exchange mechanism in consequence of the largely reduced venous oxygen saturation possibly enhanced by a decrease in blood flow. A similar pattern of oxygen saturation changes like in our study has been shown in healthy subjects during a moderate experimental increase of intraocular pressure for a maximum of 5 min insofar as oxygen saturation significantly decreased in retinal veins and tended to be reduced in retinal arteries (O'Connell et al. 2015). After IVI, there is an immediate increase in intraocular pressure, which usually normalizes not earlier than after 20 min and sometimes stays highly elevated for more than 1 hr (Bracha et al. 2018). The elevated intraocular pressure during the first minutes after the injection may therefore considerably reduce ocular perfusion pressure. Hence, the reduction of oxygen saturation in retinal vessels shortly after IVI may be more likely in consequence to a reduction in ocular perfusion pressure than an immediate pharmacological effect of the anti‐VEGF treatment.

A reduction in ocular perfusion pressure can lead to a decrease in retinal blood flow in healthy eyes if the limit of retinal blood flow autoregulation is exceeded (Riva et al. 1986). Reduced retinal blood flow would increase the time of oxygen exchange and consequently augment oxygen extraction. A reduction in choroidal blood flow during raised intraocular pressure could additionally lead to more oxygen being extracted from retinal vessels. Such changes in ocular blood flow would therefore explain the results seen in our patients. However, the study of O'Connell et al. we mentioned above not only reported an increase in AV difference during the phase of moderately raised intraocular pressure but also showed that retinal blood flow was not diminished, whereas pattern electroretinogram was significantly disturbed. This indicates that already during moderate elevation of intraocular pressure in healthy eyes there is an increased oxygen consumption of stressed retinal ganglion cells and neurons suggesting an increased metabolic demand alongside unaltered retinal perfusion (O'Connell et al. 2015). During an acute decrease of ocular perfusion pressure, oxygen extraction has been proposed to play an important role to maintain retinal function and the oxygen extraction rate per unit blood flow can increase to compensate for mild ischaemia and to maintain cellular metabolism (He et al. 2013). In addition, ranibizumab and aflibercept alone have shown in vitro a temporary increase of basal respiratory rate, ATP turnover and mitochondrial respiratory capacity (Sheu et al. 2015). An increase in AV difference shortly after IVI may therefore rather not only indicate reduced blood flow due to elevated intraocular pressure and possibly incipient anti‐VEGF hemodynamic effects but may reflect the increased oxygen metabolism under such conditions. CNV eyes showed a 15% increase in AV difference after IVI, whereas this change was much lower and not significant in eyes with DME. This different behaviour in diabetic eyes is somewhat surprising. Retinal blood flow autoregulation during experimentally induced changes in ocular perfusion pressure has been shown to be impaired in diabetes (Rassam et al. 1995) and the autoregulatory capacity of retinal perfusion further diminishes with progression of diabetic retinopathy (Grunwald et al. 1992; Mandecka et al. 2007). Assuming a more prominently reduced blood flow in diabetes due to diminished blood flow autoregulation, one would rather expect a more pronounced increase in AV difference. It seems unlikely that in diabetic retinopathy, the condition which is known to be more prone to disturbed autoregulation, blood flow changes generally have less influence on oxygen metabolism. However, the physiological coupling of intraocular pressure, ocular vascular perfusion and oxygen metabolism is complex and differs under pathological vascular conditions such as diabetic retinopathy (Hayreh 2001; Pournaras et al. 2008). Considering the previously discussed mechanisms, the abolished increase in oxygen consumption in diabetic eyes after IVI more likely indicates a limited capability of oxygen distribution due to reduced tissue perfusion and a less pronounced increase of metabolic demand due to neuroretinal tissue loss. Alternatively, neurons of the diabetic retina could be more susceptible to mechanical and pharmacologically induced stress and exhibit a reduced capacity of oxygen homoeostasis. To get more insight into the coupling mechanisms in the diabetic retina during decreased ocular perfusion pressure, it would be interesting to investigate retinal blood flow, oxygen extraction and inner retinal function during a controlled increase in intraocular pressure in diabetes patients. Unfortunately, such information is not yet available.

Albeit not statistically significant, arterial diameters in DME patients were lower than in patients with CNV. This goes in line with another large oximetry study which found reduced calibres of retinal arteries in more advanced diabetic retinopathy (Dong et al. 2016). After IVI the arterial diameter decreased only in DME patients by 6%. We attribute this to a reduced autoregulatory response in eyes with diabetic retinopathy to the reduced ocular perfusion pressure during the first minutes after injection. In healthy eyes with normal retinal autoregulation, the decrease of ocular perfusion pressure during a moderate experimental increase of intraocular pressure induces a small but continuous dilation of retinal arteries (Nagel & Vilser 2004) while blood flow slightly increases (O'Connell et al. 2015). In patients with diabetic retinopathy autoregulative responses of retinal vessel diameters and blood flow to changes in ocular perfusion pressure are known to be impaired. Previous studies showed an abnormal vasodilation in diabetes during an increase in ocular perfusion pressure, which was more pronounced in patients with retinopathy and higher glucose levels (Rassam et al. 1995; Frederiksen et al. 2006; Bek et al. 2008). Conversely, an abnormal vasoconstriction of vessel diameters in diabetes during a decrease in ocular perfusion pressure seems plausible. The exact cause of this functional change has still to be proven. Abnormal diameter reactions in diabetes during changes in perfusion pressure have been attributed to a lack of vascular tone and changes in the vessel walls. In addition, endothelial dysfunction may contribute to an imbalance of diameter regulation in diabetes (Pemp et al. 2009). Recent evidence also suggests that retinal glial cells are capable of sensing the reduction in perfusion pressure and contribute to the maintenance of vessel diameters (Li et al. 2017). Since glial cells are affected early in the diabetic retina and continue to degenerate along with retinal ganglion cells (Coughlin et al. 2017), neuroretinal damage may be another important factor in disturbed retinal vascular autoregulation.

If vessel diameters decrease under conditions of elevated intraocular pressure, retinal blood flow also decreases (Tan et al. 2017). Although blood flow was not measured directly, our results therefore indicate a reduced retinal blood flow in eyes of diabetes patients shortly after an IVI. We did not find a short‐term change in retinal vessel diameters of AMD patients. Their autoregulatory capacity therefore may have better compensated for the change in ocular perfusion pressure and retinal blood flow may not have reduced as in DME eyes at the time of measurement after IVI. The elevated AV difference in CNV eyes shortly after IVI may therefore not represent a sign of disturbed blood flow regulation in CNV eyes, similar to the findings in healthy eyes during a moderate elevation of intraocular pressure (O'Connell et al. 2015). Previous experimental data show an effect of VEGF on vascular permeability and conductance already after 20 min, but only when applied intravenously (Tilton et al. 1999). Whether similar retinal blood flow changes are also seen after VEGF depletion by the means of an IVI remains to be shown. However, shifted and superimposed effects of oculo‐hemodynamic changes during the first minutes after anti‐VEGF IVI seem possible (Mursch‐Edlmayr et al. 2019).

Reduced diameters of retinal vessels after intravitreal anti‐VEGF treatment have been shown previously in eyes with different pathologies. However, these studies investigated changes in retinal vessel diameters only one week to month after single or monthly repeated IVI, when intraocular pressure was not altered anymore. Most of the studies observed a long‐term reduction in retinal artery diameter (Soliman et al. 2008; Papadopoulou et al. 2009; Sacu et al. 2011; Corvi et al. 2015; Kurt et al. 2017; Consigli et al. 2018; Tetikoğlu et al. 2018). Tatlipinar et al. repeatedly measured retinal vessel diameters of DME and CNV eyes before and during the first week after IVI with bevacizumab. Although the reported changes were not statistically significant, arterial diameters tended to be higher at day 1 and lower at day 7 compared to baseline values (Tatlipinar et al. 2012). Taken together these results indicate a pharmacological vasoconstrictor effect of anti‐VEGF treatment on retinal vessel diameter during the first week but not immediately after IVI. Our observation that arterial diameters changed only in eyes with DME and not in eyes with CNV also point against a rapid effect of anti‐VEGF agents on retinal vessel diameter.

Previously reported values of retinal vessel diameters from oximetry measurements in subjects with and without diabetes were overall lower than in our study (Blair et al. 2017). Because of the applied oximetry analysis protocol, the number of vessels per eye accounting for our mean diameter results was different for each eye, thus a direct comparison of our baseline data with other published values may be misleading.

The main strength of our study lies in the short time passing after the injection until the second performed measurement. This provides new insights about vascular responses and oxygenation changes shortly after intravitreal injections. The short time between measurements in most of the patients also makes it unlikely that changes in retinal vascular parameters were influenced by changes in blood glucose. One limitation of our study lies in its nonrandomized design and the lack of a control group. Patients treated with placebo or healthy controls could not be included due to ethical reasons. Due to the exploratory character of the study, p‐values were not corrected for testing multiple outcomes. The p‐values and confidence intervals have to be interpreted accordingly. Additionally, a statistical model accounting for individual values and changes of intraocular pressure, blood pressure and pulse oximetry would have been more concise.

To conclude, we show that the Oxymap device enables to detect immediate changes in retinal vessel oxygenation and diameter after an IVI of anti‐VEGF agents. The observed changes can be interpreted as signs of increased metabolic demand during the physiological stress shortly after an IVI. Diabetic eyes with DME did not equally compensate for the acute alteration after IVI compared to eyes with CNV. They showed an abnormal arterial constriction and an abolished increase in AV difference. Our results indicate an impairment of vascular autoregulation and oxygen distribution and a reduced neuroretinal metabolism in the diabetic retina with a significant impact on inner retinal oxygen consumption shortly after IVI.
